# Postoperative Urinary Catheterization in Children Treated with or without Epidural Analgesia after Orthopedic Surgery: A Retrospective Review of Practice

**DOI:** 10.3390/children9091316

**Published:** 2022-08-29

**Authors:** Yotam Lior, Shimon Haim, Idan Katz, Barry Danino, Yuval Bar-Yosef, Margaret Ekstein

**Affiliations:** 1 Divisiona of Anesthesia, Intensive Care, and Pain Medicine, Tel-Aviv Sourasky Medical Center, Sackler Faculty of Medicine, Tel Aviv University, Tel Aviv 6423906, Israel; 2 Department of Pediatric Orthopedic Surgery, Dana Dwek Children’s Hospital, Tel-Aviv Sourasky Medical Center, Sackler Faculty of Medicine, Tel Aviv University, Tel Aviv 6423906, Israel; 3 Department of Pediatric Urology, Dana Dwek Children’s Hospital, Tel-Aviv Sourasky Medical Center, Sackler Faculty of Medicine, Tel Aviv University, Tel Aviv 6423906, Israel

**Keywords:** urinary catheter, epidural analgesia, postoperative urinary retention, lower extremity orthopedic surgery, neuromuscular disease

## Abstract

Epidural analgesia is effective and an accepted treatment for postoperative pain. Urinary retention is a known complication, but its description is mostly in the adult literature. Management of urinary catheter (UC) placement and removal is an important consideration in children receiving epidural analgesia. This is a single-center, retrospective observational study which examined UC management in children undergoing lower extremity orthopedic surgery under general anesthesia with or without epidural analgesia from January 2019–June 2021. Of 239 children included, epidural analgesia was used in 57 (23.8%). They were significantly younger and had more co-morbidities. In total, 75 UCs were placed in the OR, 9 in the ward, and 7 re-inserted. UC placement in the epidural group was more common (93% vs. 17%, *p* < 0.001) and remained longer (3 days vs. 1 day, *p* = 0.01). Among children without intra-operative UC, ward placement was more common in the epidural cohort (60% vs. 1.6%, *p* = 0.007). OR UC placement and ward re-insertion were more common in children with neuromuscular disease (61% vs. 22%, *p* < 0.001), (17% vs. 3%, *p* = 0.001), respectively. Based on these findings, we hypothesize that it is justifiable to routinely place a UC intra-operatively in children who undergo hip or lower extremity surgery and are treated with epidural analgesia, and caution is advised before early UC removal in orthopedic children with NMD.

## 1. Introduction

Epidural analgesia is effective and a well-accepted treatment for postoperative pain in adults and children [1,2]. Its value is well established after pediatric spine, hip, and lower extremity surgery [3,4] and is encouraged by enhanced recovery after surgery (ERAS) protocols for pediatric orthopedic surgery [5]. Recent large reviews of regional analgesia in children focus on major adverse events such as neurologic and infectious complications or systemic toxicity [6,7] and have demonstrated overall safety of this technique. Postoperative urinary retention (POUR), a known complication of epidural analgesia, is not the focus of these reviews. Its incidence and risk factors are examined mostly in the adult population [8,9,10]. While several papers have described the incidence of POUR in children to range from 4.5% to 6.3% and as high as 20% in some populations [11,12], there is a paucity of literature addressing risk factors for postoperative urinary catheterization or re-catheterization in children after surgery. Given that the latter is an invasive procedure associated with increased risks of infection, trauma, and discomfort upon insertion when not done under general anesthesia [4], it is clear that optimization of management of urinary catheter (UC) placement and removal in this population is important. Still, no evidence-based guidelines are available; rather, expert opinion dictates practice. For example, the New York School of Regional Anesthesia (NYSORA), one of the world’s leading authorities in regional anesthesia, reported a urinary retention prevalence of up to 20% in children receiving epidural continuous infusions of bupivacaine with an opioid [3]. While this report gives recommendations as to treatment of urinary retention should it occur, precise guidelines as to indications and timing for UC placement or removal in this population are still evolving. Placement of a UC in the operating room in our institution is left to the discretion of the anesthesiologist. Until this review of our practice, there were no detailed departmental guidelines as to what characteristics of the patient, surgery, or anesthetic would indicate the necessity for intra-operative urinary catheterization in children undergoing orthopedic surgery.

The primary aim of this study was to evaluate the prevalence of UC placement in the OR and in the ward, either primary placement or re-insertion after earlier removal, of a UC in children undergoing elective lower extremity orthopedic surgery under general anesthesia treated with or without epidural analgesia.

The secondary study aim was to examine the duration of UC use in the various populations.

Ultimately, the objective was to promote informed practice standards of UC placement and management in children treated with epidural analgesia after orthopedic surgery.

## 2. Materials and Methods

This is a single-center, retrospective observational study reviewing the post-operative course of consecutive children after orthopedic surgery of the lower extremity with and without epidural analgesia regarding their UC management. The study was conducted according to the guidelines of the Declaration of Helsinki and was approved by the Institutional Review Board of Tel-Aviv Medical Center (IRB No.21-0764 -TLV), which also waived the need for individual informed consent.

### 2.1. Study Population and Data Collection

Data for this study were retrieved from the hospital’s electronic records, including the anesthesia record-keeping systems, the post-anesthesia care unit, and the ward patient record systems. The study population included all surgical pediatric (<18 years) patients admitted to the pediatric orthopedics department in the Tel-Aviv Medical Center (TLVMC) following hip or lower extremity surgeries between January 2019 and December 2021.

Patients were excluded if they had emergency surgery, spinal cord anomalies from trauma or congenital deformities that caused neurogenic bladder, or if they required repeated or indwelling urinary catheterization for any reason. Recorded data included demographic and morphometric variables (gender, age, weight), American Society of Anesthesiologists’ Physical Status (ASA-PS) scores, presence of neuromuscular disease (NMD), type and length of surgery, volume of fluid and/or blood infused during surgery, and hospital length of stay.

In this cohort, NMD included children with, by descending frequency, cerebral palsy, neurofibromatosis type 1, achondroplasia, RETT syndrome, dystonia associated with congenital disorder of glycosylation type 2, epilepsy, spinal muscular atrophy type 2, spastic tetraplegia, and West-syndrome.

We recorded the type of anesthesia and whether there was placement of a continuous epidural catheter for postoperative analgesia and duration of its use. UC data included placement in the operating room (OR) or in the ward and duration of UC use. UC placement in the ward or re-insertion after removal perioperatively was considered a complication of POUR or failed trial without a catheter. 

### 2.2. Statistical Analysis

The preferred method of analysis for continuous variables was parametric using Student’s *t*-test. The non-parametric Mann–Whitney test was used if parametric assumptions could not be satisfied. Parametric assumptions were assessed using a normal plot or Shapiro–Wilks statistic for verification of normality and Levene’s test for verification of homogeneity of variances. Categorical variables were tested using Pearson’s χ^2^ test for contingency tables or Fisher’s exact test, as appropriate.

All statistical tests and/or confidence intervals, as appropriate, were 2-sided and performed at α = 0.05. All *p*-values reported were rounded to three decimal places. The data were analyzed using IBM SPSS Statistics software (version 25).

## 3. Results

During the study period, a total of 239 children (aged two months to 18 years) underwent orthopedic surgery of the lower extremities, including hip, femur, or tendon surgery at the TLVMC. Of these, 57 (23.8%) were treated with epidural analgesia. The cohort treated with epidural analgesia was significantly younger, weighed less, had higher ASA-PS scores, longer surgery, and were given more IV fluid and were more likely to have NMD, as shown in Table 1.

Data on urinary catheter (UC) management are presented in Table 2. In total, a UC was placed in the OR in 75 children, in the ward in an additional nine children, and re-inserted in the ward in seven children. Based on a comparison of epidural-treated to non-epidural treated children, overall UC placement in the epidural group was more common (93% vs. 17%, *p* < 0.001) and with longer duration (median three days vs. one day, *p* = 0.01). Of the children who did not have a UC placed intraoperatively, ward placement was more common among the epidural cohort (6/10 vs. 3/154, *p* = 0.007). However, there was no difference in UC re-insertion after UC removal (3/53 vs. 4/31) (*p* = 0.36).

A sub-analysis of data regarding children with or without NMD showed that UC placement in the OR was significantly more common in children with NMD compared to children without NMD (61% vs. 22%, *p* < 0.001). Ward re-insertion, presumed to be failure of a trial without a catheter, was also significantly more common in children with NMD compared to those without NMD (6/36 vs. 1/48, *p* < 0.001). The duration of UC was longer in NMD compared with non-NMD patients (median three days vs. one day, *p* = 0.003). Epidural catheter duration was comparable between groups with and without NMD.

## 4. Discussion

We performed a retrospective, single-center study of UC management in 239 pediatric patients who underwent elective orthopedic surgery of the lower extremity between January 2019 and June 2021. Our main findings were that children with epidural analgesia are treated with intra-operative UC more frequently and for a longer postoperative period compared to children without epidural analgesia (82% vs. 15%, *p* < 0.001, and median three days vs. one day, *p* = 0.01, respectively). Furthermore, among children without intra-operative UC placement, ward UC placement was more frequent in the epidural group (6/10 vs. 3/154, *p* = 0.007). We also found that children who were treated with epidural analgesia comprised a population with a higher load of comorbid conditions. When children with neuromuscular disease were analyzed separately, we found that they had significantly longer UC durations (median three days vs. one day, *p* < 0.003); more importantly, they were more likely to undergo-re-catheterization in the ward postoperatively after UC removal (17% vs. 3%, *p* = 0.001).

A novelty of our study is that it specifically addresses the complication of ward UC placement in a large series of children undergoing orthopedic surgery.

These findings are consistent with recent publications regarding the overall POUR rate in children of 5% [11,12]. Our finding of longer UC duration postoperatively and higher incidence of UC re-insertion in the NMD population is consistent with that of Buckon’s [13] and Brenn’s [14] review of UC management in post-orthopedic surgery in children with cerebral palsy (CP). It is important to note, however, that re-insertion rates differ significantly between the studies (Buckon: 9.2%, Brenn: 70%).

While both general and regional anesthesia have previously been implicated as contributing factors in the occurrence of POUR in children [13], current literature is still sparse regarding the occurrence of POUR in children treated with epidural analgesia [6,7]. Furthermore, recent publications on regional anesthesia in children focus mostly on major adverse events such as neurologic, infectious, or systemic toxicity in children treated with epidural analgesia, but do not emphasize the risks of POUR and/or peri-operative UC placement.

Ward urinary catheterization in children has potentially harmful effects, including stress and discomfort, which may require additional sedation, possibility of infection, and rarely acute kidney injury because of urinary outflow obstruction [4]. While the risks of ward UC placement and re-placement have been described, no guidelines regarding indication for UC placement and UC management in children following orthopedic surgery after general anesthesia with or without epidural analgesia are available.

In the absence of clear guidelines, our observations of frequent UC ward placement in epidural-treated children support the practice of mandatory intraoperative UC placement in this population.

Given accumulated evidence of potential risks associated with longer duration of UC use [15], early removal of UC was recommended as early as 1988 in adults following orthopedic, abdominal, and thoracic surgery [9,16] while still receiving epidural analgesia. Our data suggest that UC and epidural use durations are often similar. One possible explanation for this finding is that UCs were removed in the ward at the same time or following epidural catheter removal as part of the clinical departmental routine. Future prospective studies should assess the outcome of early removal of intraoperatively placed UC in selected pediatric epidural patients [16] while still using the epidural catheter, allowing effective postoperative analgesia in children after orthopedic surgery.

Lastly, our data suggest the need for a specialized UC management approach in children with NMD following orthopedic surgery. While our observations suggest that a greater degree of caution in UC removal is already applied (as reflected by the longer durations of UC use), high rates of UC re-insertions suggest that these children are at the highest risk for ward UC re-insertion.

Our study has several limitations. The major limitation of this study is its retrospective design with its inherent potential for unmeasured confounding factors as well as the relatively low rates of POUR requiring ward UC insertion. Another significant limitation of this study relates to the manner in which POUR was diagnosed. While ideally POUR will be diagnosed using urinary bladder ultrasound assessment, the diagnosis in our cohort was performed based on a clinical presentation. Patients not urinating spontaneously 12 h after UC removal were examined; if the urinary bladder was palpated, UC was re-inserted. Therefore, we limit our discussion to the complication of ward placement or re-insertion of a UC but cannot verify that all of these events truly represent POUR.

## 5. Conclusions

Intraoperative as well as ward UCs are placed more frequently in patients with epidural analgesia failure of a trial without a catheter is more common in children with NMD. Based on these findings, we hypothesize that it is justifiable to routinely place a UC intra-operatively in children who undergo hip or lower extremity surgery and are treated with epidural analgesia, and caution is advised before early UC removal in orthopedic children with NMD. Future prospective large-scale studies with ultrasound assessment of urinary retention would further advance our understanding of criteria for UC placement in this population.

## Figures and Tables

**Table 1:** Characteristics of children undergoing orthopedic surgery of hips or lower extremities with and without epidural analgesia.

	Total Population (*n* = 239)	Non-Epidural (*n* = 182)	Epidural (*n* = 57)	*p*-Value
Age at operation, years	9.2 (5.0)	9.5 (5.1)	8.1 (4.3)	0.03
Age < 1 year	20 (8.7%)	20 (12%)	0 (0%)	0.005
Gender, female	101 (42.4%)	77 (42.5%)	24 (42.1%)	0.95
Weight, kg	35 (24)	38 (26)	25 (16)	0.001
ASA-PS score	2 [1, 2]	2 [1, 2]	2 [2, 3]	0.005
Neuromuscular disease ^a^	57 (24%)	36 (20%)	21 (37%)	0.008
Intraoperative crystalloid administration, mL/kg	27 (28)	22 (25)	43 (31)	<0.001
Intraoperative packed cells transfusion y/*n*	10 (4%)	6 (3%)	4 (7%)	0.26
Length of surgery, hours	2.5 [1.1–3.5]	1.5 [1.0, 2.8]	3.6 [2.7, 4.1]	<0.001

Baseline and intraoperative characteristics of included patients, according to use of epidural analgesia. Data are presented as the means (SD), N (%), or median [IQR] as appropriate. ASA-PS, American Society of Anesthesiologists physical status; IQR, interquartile range; SD, standard deviation. ^a^ Includes by descending frequency: Cerebral palsy, Neurofibromatosis type 1, Achondroplasia, RETT syndrome, Dystonia associated with congenital disorder of glycosylation type 2, Epilepsy, Spinal muscular atrophy type 2, Spastic tetraplegia, West-syndrome.

**Table 2:** Urinary catheter management of children undergoing orthopedic surgery of the hips or lower extremities with and without epidural analgesia.

Variable			Total Population (*n* = 239)	Non-Epidural (*n* = 182)	Epidural (*n* = 57)	*p*-Value	Non-NMD ^a^ (*n* = 182)	NMD ^a^ (*n* = 57)	*p*-Value
UC placement	No		155 (65%)	151 (83%)	4 (6%)	<0.001	134 (73%)	21 (37%)	0.004
	Yes	OR	75 (31%)	28 (15%)	47 (82%)	<0.001	40 (22%)	35 (61%)	<0.001
		Ward	9 (4%)	3 (2%)	6 (11%)	0.007	8 (4%)	1 (2%)	0.69
UC re-insertion in surgical ward			7 (3%)	4 (2%)	3 (5%)	0.36	1 (3%)	6 (17%)	0.001
Duration of UC use, days (median [IQR])			2 [1, 3]	1 [0, 2]	3 [1, 3]	0.01	1 [0.75, 3]	3 [2, 3]	0.003
Duration of epidural use, days (median [IQR])			3 [2, 3]	n/a	3 [2, 3]	n/a	3 [2, 3]	3 [3, 3]	0.06

Characteristics of urinary catheter use in children with or without epidural and children with and without neuromuscular disease. Data presented as *n* (%) unless stated otherwise. UC, urinary catheter; IQR, interquartile range; OR, operating room; NMD, Neuromuscular disease. ^a^ Includes by descending frequency: Cerebral palsy, Neurofibromatosis type 1, Achondroplasia, neuroblastoma, RETT syndrome, Dystonia associated with congenital disorder of glycosylation type 2, Epilepsy, Spinal muscular atrophy type 2, Spastic tetraplegia, West-syndrome.

## Data Availability

Data supporting reported results from this study can be found in secure computers in the medical center.
